# Sleep apnea and its role in transportation safety

**DOI:** 10.12688/f1000research.12599.1

**Published:** 2017-12-22

**Authors:** Maria Bonsignore

**Affiliations:** 1 DiBiMIS, University of Palermo, Palermo, Italy; 2 CNR Institute of Biomedicine and Molecular Immunology (IBIM), Palermo , Italy

**Keywords:** Obstructive Sleep Apnea, Sleep disorders, CPAP, driving accidents

## Abstract

Obstructive sleep apnea (OSA) is a main cause of excessive daytime sleepiness and increases the risk for driving accidents, which can be normalized by treatment with continuous positive airway pressure ventilation. Since it is estimated that OSA is not diagnosed in about 80% of cases, recognition of patients at risk for driving accidents is a problem from both medical and societal points of view. Strategies to screen and identify subjects at high risk for driving accidents are under study in order to improve safety on the road, especially for commercial drivers, who show a high prevalence of OSA.

OSA is characterized by partial (hypopnea) or complete (apnea) collapse of upper airway during sleep, causing intermittent hypoxemia and sleep fragmentation. Respiratory pauses during sleep referred by the partner, intermittent snoring, and excessive daytime sleepiness (EDS) are the most frequent OSA symptoms, but the clinical picture may vary. Subjective EDS is reported by only 50% of patients with OSA ^1^. OSA severity is defined as the number of respiratory events per hour of sleep (apnea-hypopnea index, or AHI). Mild, moderate, and severe OSA are defined on the basis of AHI cutoffs of 5 to less than 15, 15 to less than 30, and 30 or more, respectively ^1^.

OSA is the most frequent type of sleep-disordered breathing in the adult general population. OSA is often associated with obesity but can occur in normal-weight subjects. Both anatomical and functional factors contribute to the pathogenesis of upper airway collapse during sleep ^2^. OSA prevalence increases with age and body mass index and is higher in men than in women. According to a recent meta-analysis, the prevalence of OSA in the general population ranges between 9% and 38% for an AHI cutoff of at least five events per hour ^3^. The HypnoLaus study in over 2,000 subjects from the general population in Lausanne reported much higher prevalences of moderate to severe OSA (AHI ≥15 per hour): 23.4% in women and 49.7% in men ^4^.

The gold standard of OSA treatment is continuous positive airway pressure (CPAP) during sleep, manually or automatically titrated to the therapeutic level in each patient. Application of CPAP prevents upper airway collapse and all the health consequences of OSA ^1^. Adherence to CPAP treatment, however, is often suboptimal, especially in patients with mild or no OSA symptoms. Currently, the use of CPAP for at least 4 hours per night for 70% of nights is considered evidence of good adherence ^5^, although more prolonged use is necessary to prevent the full spectrum of OSA-associated derangements ^6^. Other treatment modalities are available. Mandibular advancement devices are often preferred by patients but are more effective in mild to moderate than in severe OSA ^7^. Upper airway surgery is an additional option, but the success rate is highly variable ^8^. In obese patients, lifestyle interventions and bariatric surgery are associated with a reduction of AHI, although a complete resolution of OSA may be difficult to achieve ^9^.

OSA is often associated with systemic hypertension and cardiovascular comorbidities. Severe untreated OSA increases the risk for all-cause and cardiovascular mortality ^10^. Data from the Sleep and Stent Study, a longitudinal observational study in patients undergoing percutaneous coronary interventions, showed an independent association between OSA and incidence of major adverse cardiac and cerebrovascular events during follow-up ^11^. However, recent randomized controlled trials failed to confirm any significant effect of CPAP treatment in minimally symptomatic patients with coronary artery disease and OSA ^12, 13^. A recent meta-analysis found a significant protective effect of CPAP only in patients who used CPAP for more than 4 hours per night ^14^. According to these data, CPAP treatment might be indicated more for control of symptoms than for prevention of OSA-associated risk, since low compliance to CPAP treatment remains a problem in asymptomatic patients.

EDS is associated with increased risk of motor vehicle accidents and a high rate of fatal accidents ^15^. OSA is not the only cause of EDS, since sleep deprivation and shift work also contribute to sleepiness at the wheel. A survey promoted by the European Sleep Research Society (over 12,000 questionnaires obtained from 19 countries) reported the alarming result that 17% of respondents had fallen asleep at the wheel in the previous 2 years ^16^. Individual factors predicting sleepiness at the wheel were younger age, male gender, driving at least 20,000 km per year, higher EDS, and high risk for OSA assessed by questionnaire. Compared with controls, patients with OSA show a 2.5-fold risk for car accidents ^17^ and occupational accidents ^18, 19^. In the European Sleep Apnea Database (ESADA) cohort, driving risk increased with OSA severity; interestingly, obesity, short sleep time, and younger age were associated with high driving distance per year ^20^. A longitudinal study on driving accidents, based on data derived from the Sweden Traffic Accidents Registry, found that OSA severity did not predict accidents, and significant risk factors were driving distance, EDS, habitual sleep shorter than 5 hours per night, and use of hypnotics. In patients with OSA, treatment with CPAP with good adherence was found to normalize the risk for accidents ^21^.

Although the role of OSA in driving accidents has been known for about 20 years, it has regained attention recently because of the need to increase safety on the road. In 2014, a European Union (EU) regulation for the first time set the requirements for issuing a driver’s license to patients with moderate to severe OSA ^22^. Since EDS and driving risk in OSA can be reversed to normal after treatment with CPAP ^23, 24^, the EU Directive mandates objective assessment of compliance to CPAP in treated OSA patients. A driver’s license can be issued to patients with objectively documented good adherence to CPAP treatment, and reassessment of fitness to drive and compliance to treatment is planned at 3-year intervals in noncommercial drivers and after 1 year in commercial drivers ^22^. However, symptomatic cases represent only a small fraction of the subjects with significant sleep-disordered breathing, since comparison between epidemiological data and actually identified OSA patients indicates that OSA remains undiagnosed in about 80% of cases.

The attitude for management of OSA patients with regard to issuing a driver’s license is highly variable. A survey of British doctors highlighted large disagreement in the evaluation of sleepiness and driving risk in patients with OSA and variable levels of acceptable compliance to CPAP treatment with regard to driving risk ^25^. In addition, the medical personnel in charge of the evaluation varies in different countries, from the pulmonologist to the sleep specialist or the personnel of the national agency issuing the driver’s license. In summary, a clear model applicable in a standardized way in different countries is still lacking, and its implementation will require a considerable educational effort to instruct the health service personnel about screening for OSA and identifying patients potentially at high risk for accidents.

The issue of safety is particularly relevant in commercial drivers, who show a higher prevalence of OSA (between 28% and 78%) compared with the general population and are more exposed to risk of driving accidents because of their high mileage per year compared with noncommercial drivers ^26^. Accordingly, commercial drivers should be subjected to tighter control of their health status, including OSA screening and assessment. The American Academy of Sleep Medicine has issued a comprehensive and detailed document on screening and diagnostic and therapeutic approaches for commercial drivers ^26^. This document underlines the cost-effectiveness of OSA treatment and the substantial savings associated with CPAP treatment and the related prevention of drowsiness-related accidents. Moreover, OSA treatment improved the amount and quality of sleep, quality of life, and overall health costs ^26^.

A recent study reported the effect of a screening program promoted by the transportation industry with the aim to detect and treat OSA in commercial drivers and monitor the frequency of accidents according to adherence to treatment ^27^. This study confirmed a high risk associated with untreated severe OSA in commercial drivers (that is, a fivefold risk in drivers with OSA nonadherent to CPAP treatment compared with controls) and the protective effect of CPAP treatment (that is, similar accident rate in non-OSA and CPAP-adherent drivers with OSA) ^27^. Nevertheless, only 46% of drivers with OSA on CPAP showed good adherence to treatment. This longitudinal study, the first specifically focused on commercial drivers, raised a number of questions on the best strategy to improve driving safety and, as underlined in the accompanying editorial ^28^, highlights the difficulties in developing a feasible set of legal rules to be applied in daily practice on a large scale to avoid a major risk for humans and significant property damage.

OSA represents a relevant risk for car accidents because of its rising prevalence in the population in association with the obesity epidemic. Unlike alcohol drinking, which can be readily identified by the police through an immediate measurement of the blood alcohol level, sleepiness of drivers at the wheel is impossible to identify in a straightforward way. Therefore, a screening strategy can be implemented only when the driver’s license is issued or renewed, as indicated by the EU Directive; this document, however, does not indicate any specific protocol to be followed, and national regulations have to be developed and implemented by each member state. To date, only the Spanish Sleep Society in Europe has published an updated protocol to address the issuing or renewing of drivers’ licenses according to the EU Directive ^29^.

The current screening strategy relies on validated questionnaires to identify subjects at risk for OSA requiring further investigation. A recent meta-analysis compared sensitivity, specificity, and diagnostic odds ratio of the Berlin, STOP-Bang, STOP, and Epworth Sleepiness Scale (ESS) questionnaires ^30– 33^, all based on clinical assessment of anthropometrics and symptoms. The ESS and the STOP-BANG questionnaire showed the highest sensitivity ^34^. Data from the HypnoLaus cohort were recently used to develop the NoSAS score based on the assessment of risk factors such as neck circumference, body mass index, snoring, age, and gender, and results were verified in an additional cohort ^35^. However, screening for OSA by questionnaires is far from perfect, since their sensitivity and specificity vary according to the population under study, the type of sleep test used for assessment, or presence of comorbidities ^36, 37^.

In clinical practice, subjective EDS is usually evaluated by the ESS, a questionnaire administered to the patient on the likelihood of falling asleep in any of eight situations, according to a score of 0 to 3 (from unlikely to very likely) ^33^. Subjective EDS is considered to be present if the ESS score is at least 10 and severe if ESS is at least 16. The ESS score is a good estimate of EDS in symptomatic OSA patients but cannot be considered totally reliable. For example, the need to renew a driver’s license and the fear of losing one’s job as a truck driver are known causes of voluntary underreporting of EDS by the patient. Therefore, objective assessment of sleepiness is needed, as is a sleep study to assess the occurrence of OSA and its severity.

Objective evaluation of EDS by the Mean Sleep Latency Test (MSLT) or Maintenance of Wakefulness Test (MWT) after nocturnal polysomnography (PSG) in the sleep laboratory is not applicable in large numbers of patients. Moreover, nowadays, OSA diagnosis is often obtained by respiratory polygraphy (that is, by monitoring cardiorespiratory signals without sleep staging, which is necessary for MSLT or MWT obtained on the day after PSG). Whereas MSLT is not very sensitive in patients with OSA and shows several limitations ^38^, MWT may better reflect the capability to maintain vigilance under monotonous driving such as on a freeway ^39– 41^, especially when prolonged trials are used (that is, 40 instead of the usual 20 minutes) ^42^.

Driving simulators might represent a good diagnostic option, but they have not yet entered clinical practice and are mostly used for research purposes. Driving simulator tests yield a considerable amount of information; for example, they can measure deviation of lane position and reaction times ^43^. Failure during simulated driving was associated with the following responses obtained in OSA patients filling out a detailed anonymous questionnaire on driving: the need to take a break after less than one hour of driving, the likelihood of feeling moderately to highly sleepy while driving, and the occurrence of nodding off while driving in the previous year ^44^. Differences in ESS between patients failing or not failing at the driving simulator test were small, confirming the known finding that referred sleepiness at the wheel might be more useful than sleepiness in general to identify OSA patients at risk of accidents ^45^. A recent systematic review comparing performance at driving simulators and occurrence of real driving accidents casts some doubt on the potential clinical impact of driving simulation to predict real accidents in the individual patient ^46^. Twelve studies, which included small samples of subjects, were analyzed and used heterogeneous protocols and different simulators, indicating a strong need for further research in this field.

Other experimental studies have investigated possible predictors of sleepiness and driving performance in patients with OSA. Spectral analysis of sleep electroencephalography (EEG) ^47^ or sophisticated analysis of EEG during wakefulness ^48, 49^ may provide sensitive markers of impaired driving performance. Eyelid movements and closure appear to be promising markers to detect sleepiness ^50– 53^ and have the advantage of being potentially used to identify a driver’s EDS inside the vehicle with the use of sensors.

In conclusion, while the problem of driving safety in general, and in patients with OSA in particular, is currently investigated in different parts of the world, we are still in an early phase, since tests to be used on a large scale to assess fitness to drive with regard to EDS are lacking. Meanwhile, road safety campaigns and education of the general public about sleepiness at the wheel should be implemented at the national level to increase awareness, similar to what already happens regarding cell phone use or texting while driving, which are known to greatly increase the risk of car accidents ^54^. In addition, the development of new technologies will likely improve vehicle safety by the use of sensors to alert the sleepy driver. OSA-related accidents can be prevented by OSA treatment in the large majority of cases, but patients seeking medical help account for only about one-fifth of OSA cases occurring in the general population, and a standardized protocol to identify previously unknown OSA cases at high risk of accidents is lacking. A joint Task Force of the European Respiratory Society and the European Sleep Research Society is currently at work to suggest the best available protocol to apply the EU Directive on Sleepiness while driving in patients with OSA and to suggest a research agenda to improve safety on the road in Europe ^55^.
